# Adolescents with obesity treated with exenatide maintain endogenous GLP-1, reduce DPP-4, and improve glycemic control

**DOI:** 10.3389/fendo.2023.1293093

**Published:** 2023-11-01

**Authors:** Rasmus Stenlid, Sara Y. Cerenius, Quan Wen, Banu Küçükemre Aydin, Hannes Manell, Azazul Chowdhury, Hjalti Kristinsson, Iris Ciba, Erik S. Gjessing, Katharina Mörwald, Julian Gomahr, Verena Heu, Daniel Weghuber, Anders Forslund, Peter Bergsten

**Affiliations:** ^1^ Department of Medical Cell Biology, Uppsala University, Uppsala, Sweden; ^2^ Department of Women’s and Children’s Health, Uppsala University, Uppsala, Sweden; ^3^ Department of Pediatric Obesity, Uppsala University Children’s Hospital, Uppsala, Sweden; ^4^ Department of Pediatrics, Paracelsus Medical University, Salzburg, Austria; ^5^ Obesity Research Unit, Paracelsus Medical University, Salzburg, Austria

**Keywords:** obesity, GLP-1, GLP-1 receptor analog, exenatide, pediatrics, glycemic control, GLP-1 receptor agonist, treatment

## Abstract

**Background:**

GLP-1 receptor agonists (GLP-1RA) are increasingly used to treat adolescent obesity. However, the effect on endogenous GLP-1 secretory patterns following treatment in adolescents is unknown. The GLP-1RA exenatide was shown to significantly lower BMI and 2-hour glucose in adolescents with obesity, in the placebo-controlled, randomized controlled trial Combat-JUDO. The aim of this study was to evaluate effects of weekly injections of 2 mg exenatide extended release on secretory patterns of endogenous hormones during OGTT.

**Subjects and Measurements:**

This study was a pre-planned sub-study of the Combat-JUDO trial, set at the Pediatric clinic at Uppsala University Hospital, Sweden and Paracelsus Medical University, Austria. 44 adolescents with obesity were included and randomized 1:1 to treatment:placebo. 19 patients in the treatment group and 18 in the placebo group completed the trial. Before and after treatment, GLP-1, glucose, insulin, glucagon and glicentin levels were measured during OGTT; DPP-4 and proinsulin were measured at fasting. A per-protocol approach was used in the analyses.

**Results:**

Exenatide treatment did not affect GLP-1 levels during OGTT. Treatment significantly lowered DPP-4, proinsulin and the proinsulin-to-insulin ratio at fasting, increased glicentin levels but did not affect insulin, C-peptide or glucagon levels during OGTT.

**Conclusion:**

Weekly s.c. injections with 2 mg of exenatide maintains endogenous total GLP-1 levels and lowers circulating DPP-4 levels. This adds an argument in favor of using exenatide in the treatment of pediatric obesity.

**Clinical trial registration:**

clinicaltrials.gov, identifier NCT02794402

## 1 Introduction

Obesity rates amongst the world’s children and adolescents increased from less than 1% in 1975 to approximately 7% in 2016 (1–3). Numbers which are expected to rapidly increase in the coming years, to affect about 208 million boys and 175 million girls by 2035 (3). Obesity is a chronic, relapsing disease, associated with numerous co-morbidities and complications (4). Some of these complications, including type 2 diabetes mellitus (T2DM), develop early in life (5). The progression of obesity takes place in a context where biological predisposition, socioeconomic, and environmental factors interact to promote initially hyperplasia and subsequently hypertrophy of the adipose tissue (6). Today, bariatric surgery is the most effective treatment for obesity in adults and adolescents (7, 8). In adolescents, surgery is only indicated for severe obesity and it is infrequently used (9). The American Academy of Pediatrics now recommends that adolescents aged ≥12 years with obesity should be offered weight loss anti-obesity medication (AOM) according to medication indications, risks and benefits, as an adjunct to health behavior and lifestyle treatment (10).

Although AOM options have been limited for the pediatric population (11), glucagon-like peptide 1 (GLP-1) receptor agonists (GLP-1RA) show significant beneficial effects on both BMI and metabolic health for the treatment of obesity in adults as well as in children and adolescents (4, 12, 13). Indeed a recent study found an impressive 16.1% decrease in BMI in adolescents with obesity using the GLP-1RA semaglutide for 68 weeks (14). Adding to this, liraglutide and semaglutide has been approved by both the FDA and the EMA for treatment of obesity from 12 years of age (6, 15, 16). However, greater weight gain has been observed in children treated with GLP-1RA shortly after discontinuation of treatment, compared to placebo (4). The reason for this is unknown, but prolonged exogenous stimulation of hormone-specific receptors may influence endogenous hormonal regulation (17). Contradictory observations have been reported regarding this phenomenon. One study noted an increase of endogenous total GLP-1 following treatment with the GLP-1RA liraglutide in adults with early T2DM (17). Conversely, liraglutide has also been shown to have the opposite effect, suppressing endogenous concentrations of total GLP-1 and other proglucagon-derived peptides (18).

A suppression of endogenous GLP-1 might contribute to the greater weight gain observed in the GLP-1RA treated group compared to the placebo group shortly after discontinuation of treatment in another trial (4). Hence, the use of GLP-1RA as a life-long treatment strategy is still a topic for debate. Nevertheless, whether this increased weight gain is really due to a suppressing effect on endogenous GLP-1 is not known. Also, it is not known whether the same suppressing effect on endogenous proglucagon-derived peptides that is seen in adults (18), is also seen in children and adolescents. Neither is it fully known if different GLP-1RA have different effects on the endogenous hormonal systems. Hence, we need to better understand the effects of treatment on physiology in the context of prolonged or even chronic treatment strategy.

In this pre-planned sub study of the Combating JUvenile Diabetes and Obesity (Combat-JUDO (13)) randomized controlled trial, we measured the effect of the GLP-1RA exenatide on endogenous hormonal regulation in adolescents with obesity. The parameters measured were glucose tolerance, endogenous secretory patterns of the proglucagon-derived hormones GLP-1, glucagon, and glicentin, as well as insulin, proinsulin, C-peptide and the enzyme dipeptidyl peptidase-4 (DPP-4).

## 2 Materials and methods

The Combat-JUDO trial was a 6-month multi-center, randomized, double-blinded, parallel, placebo-controlled trial (clinicaltrials.gov: NCT02794402) comparing once weekly subcutaneous injections of 2 mg exenatide extended release (Bydureon, AstraZeneca, Cambridge, UK) to placebo for the treatment of pediatric obesity (13). The design, protocol, and main outcomes have previously been reported in detail (13). In short, the primary outcome was change in BMI-SDS, which was significantly lowered following treatment (13). In addition, the 2h glucose value was significantly lowered following treatment (13). The trial was conducted between September 2015 and September 2016 in Uppsala, Sweden and Salzburg, Austria. Patients were included (n=44, of which female n=22) and randomized 1:1 to treatment:placebo. Nineteen patients completed the trial in the exenatide group and 18 in the placebo group. Three patients in the exenatide group and four patients in the placebo group did not complete the trial. In the exenatide group, one patient dropped out due to family reasons and two due to inappropriate handling of the study medication. In the placebo group, one patient withdrew voluntarily, two due to protocol noncompliance and one due to inappropriate handling of the study medication. Since the aim of this pre-planned study was to evaluate direct effects on biological systems induced by the treatment, a per-protocol approach (19) was deemed to more accurately provide an answer than the intention-to-treat approach to this aim. All analyses include only the patients who completed the trial according to protocol.

Inclusion criteria were age between 10 to 18 years and obesity duration > 5 months. Exclusion criteria were syndromic obesity, pregnancy, severe disease (including all forms of diabetes mellitus), abnormal QT-interval and any treatments influencing blood glucose, weight, or other parameters of the metabolic syndrome.

### 2.1 Venous blood sampling and oral glucose tolerance test

An oral glucose tolerance test (OGTT) was performed at the beginning and the end of the trial. Glucose (APL, Stockholm, Sweden) was dissolved in water according to the dosage 1.75 g/kg of body weight, with a maximum of 75 g in total. Fasting samples were drawn through a patent venous catheter, which was applied after local anesthesia (EMLA; AstraZeneca), at -15 and -5 minutes prior to the consumption of glucose. The dissolved glucose was then orally consumed by the participants and blood was subsequently sampled at 5, 10, 15, 30, 60, 90, 120, and 180 minutes. Blood samples in EDTA (for all analyses except glucagon, GLP-1 and glicentin) or P800 tubes (Becton Dickinson, Franklin Lakes, NJ) were immediately placed on ice and centrifuged at 4°C and 2500g for 10 minutes, after which plasma was aliquoted and stored at -80°C until analysis. P800 tubes contain a DPP-4 inhibitor, and blood from these tubes were used for the glucagon and GLP-1 analyses.

### 2.2 Biochemical analyses

Glucose levels were measured using a glucose oxidation method (Architect c8000 instrument, Abbott Diagnostics, Abbott Park, IL). Total GLP-1 was analyzed by a chemiluminescent enzyme-linked assay (Mercodia, Uppsala, Sweden, Catalog # 10-1278-01, RRID: AB_2892202) with, according to the manufacturer, a 100% specificity to the GLP-1 (9–36) amide. The ELISA has a reported 88% cross-reactivity with the GLP-1 (1–36) amide, 93% cross-reactivity with the GLP-1 (7–36) amide, and non-detectable cross reactivity with the glycine-extended GLP-1 (1–37) amide, the GLP-1 (7–37) amide, and exenatide. Thus, the GLP-1 assay measures uncleaved and cleaved 36 amino acid amidated species of endogenous GLP-1. Postprandially, levels of GLP-1 (7–37) are low compared to GLP-1 (7–36), hence the error of using an amidation-specific assay is small when measuring postprandial samples (20). Plasma levels of insulin (Mercodia, Catalog # 10-1113-01, RRID: AB_2877672), glucagon (Mercodia, Catalog # 10-1271-01, RRID: AB_2737304), glicentin (Mercodia, Catalog #10-1273-01, RRID: AB_2884906), proinsulin (Mercodia, Catalog # 10-1118-01, RRID: AB_2754550), and C-peptide (Mercodia Catalog # 10-1136-01, RRID : AB_2750847) were determined using ELISAs. Circulating DPP-4 was analyzed with a sandwich ELISA (R&D systems, Minneapolis, MN, Catalog # AF1180, RRID : AB_354651). Circulating plasma DPP-4 levels are shown to have a strong correlation with plasma DPP-4 enzymatic activity (21).

In order to minimize cross-reactivity with other proglucagon-derived peptides, the glucagon ELISA uses one C-terminal and one N-terminal antibody. The glicentin ELISA uses one antibody towards the glucagon sequence and one toward the glicentin-related polypeptide sequence. Hence it uses two side-viewing antibodies and has no reported cross-reactivity with glucagon, oxyntomodulin, mini-glucagon, GLP-1, or GLP-2 (5). Cross-reactivity for the insulin assay with C-peptide and proinsulin is less than 0.01%.

### 2.3 Statistical analyses

All data are presented as mean ± SEM unless otherwise specified. Differences between two groups were determined using Student’s t-test. For fasting samples, paired t-tests were used. Normality of data distribution was determined by the Shapiro-Wilk test and the examination of QQ-plots. Differences between the groups during the OGTT were determined using two-way ANOVA, differences at fasting between multiple groups were analyzed using one-way ANOVA, both followed by Tukey’s *post hoc* test, when the data followed the Gaussian distribution. For data that did not follow the Gaussian distribution, Kruskal-Wallis test followed by Dunn’s test was used. Area under curve (AUC) was calculated by the trapezoid rule with 0 as baseline. A ROUT test (Q=1%) was used to identify statistical outliers.

All statistical analyses were performed in GraphPad Prism v9.2 (GraphPad Software, La Jolla, CA). *P* values less than 0.05 were considered statistically significant.

### 2.4 Ethical considerations

The study was accepted for Voluntary Harmonization Procedure (VHP673, VHP2015061) and approved by ethics committees and regulatory authorities in Sweden and Austria (EudraCT No: 2015-001628-45; EC Sweden: Dnr 2015/279; EC Austria: 415-E/1544/20-2014). Informed consent and assent were obtained from parents and patients, respectively. The trial was conducted according to the Declaration of Helsinki (World Medical Association; Version 2013) and the E6 Guideline for Good Clinical Practice (International Conference on Harmonization).

## 3 Results

The groups did not differ at baseline regarding age, sex, BMI, BMI-SDS, pubertal status according to Tanner staging, or glucose tolerance (**Table 1**).

**Table 1:** Baseline clinical characteristics of the study group.

	Placebo (n=18)	Exenatide (n=19)	*p* value
**Age (years)**	13.1 ± 1.9	14.3 ± 2.4	0.11
**Sex (n female)**	8	11	0.42
**Tanner staging (median ± IQR)**	3.5 ± 2.5	4 ± 2	0.18
**BMI**	37.4 ± 3.1	37.5 ± 3.8	0.93
**BMI-SDS**	3.2 ± 0.4	3.2 ± 0.5	0.95
**Fasting glucose (mmol/L)**	5.3 ± 0.3	5.3 ± 0.4	0.77
**2h glucose (mmol/L)**	6.4 ± 1.0	6.4 ± 1.1	0.89

All values are presented as mean ± SD, unless otherwise stated.

**Figure 1:**
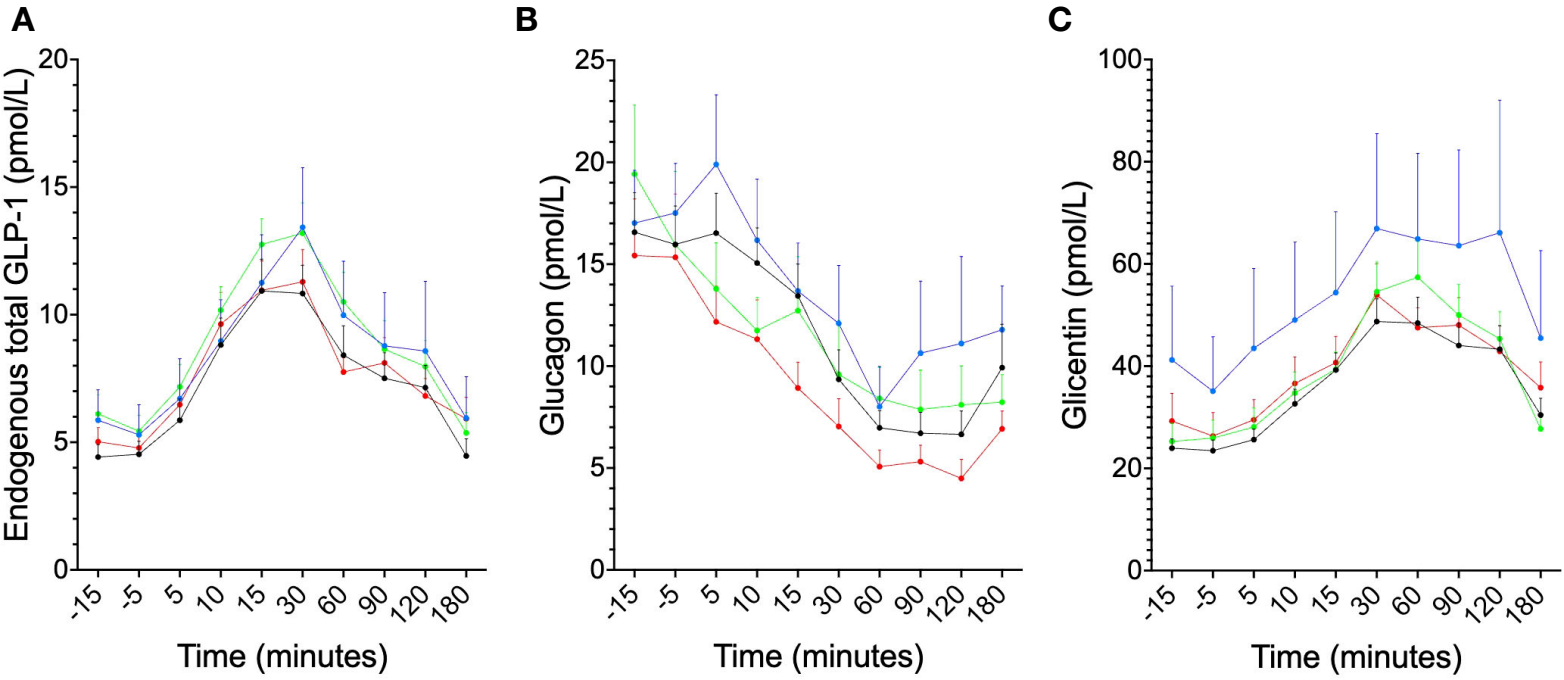
Secretory patterns during the OGTT for proglucagon-derived peptides, illustrated as: before exenatide (black), after six months of exenatide (blue), before placebo (red) and after six months of placebo (green) for **(A)** endogenous total GLP-1, **(B)** glucagon and **(C)** glicentin, (for glicentin, p<0.05 for before vs after treatment with exenatide). Means ± SEM are shown.

**Figure 2:**
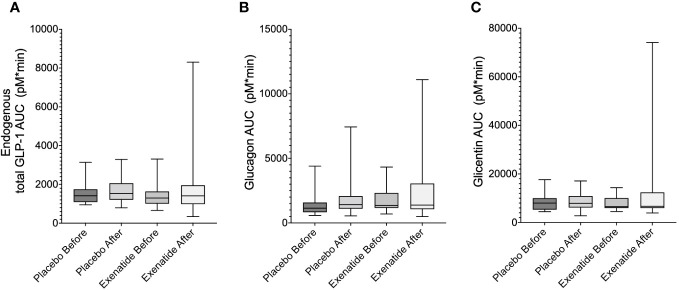
Area under the curve during the OGTT (AUC_0-180 min_) are shown for: **(A)** endogenous total GLP-1, **(B)** glucagon and **(C)** glicentin. Means ± SEM are shown.

No difference in total endogenous GLP1-levels during the OGTT was observed between the treatment groups when comparing either the curves (**Figure 1A**) or the area under the curve (AUC) (**Figure 2A**). Similarly, no differences were seen between the groups, in terms of curve (**Figure 1B**) or AUC (**Figure 2B**) of glucagon during the OGTT. Glicentin was significantly (*p*<0.05) increased after treatment when comparing curves (**Figure 1C**), but not when comparing AUC (**Figure 2C**). Notably, there was a large variability after treatment for all three hormones.

**Figure 3:**
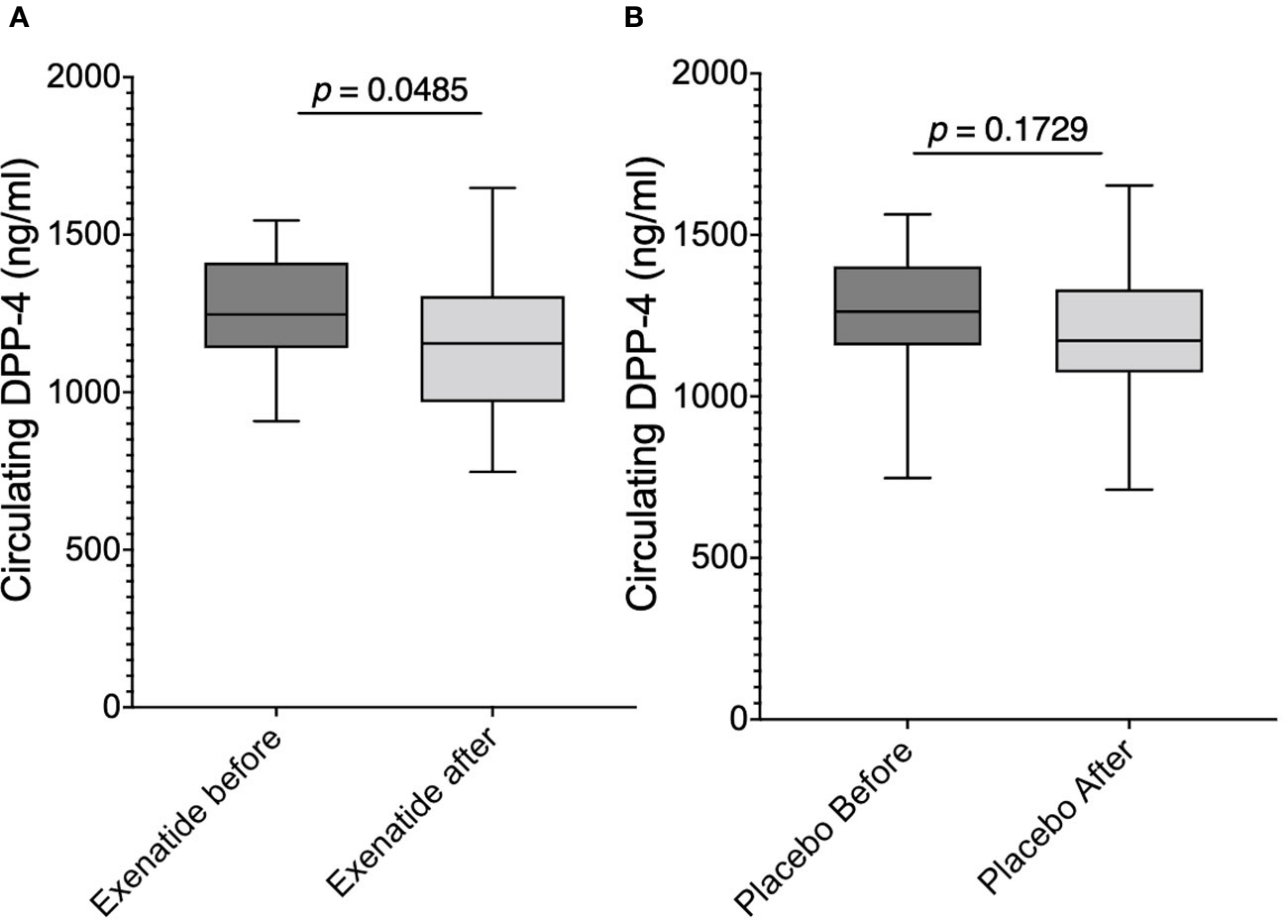
Fasting plasma levels of DPP-4 **(A)** before and after exenatide treatment, **(B)** before and after placebo. Means ± SEM are shown.

The fasting plasma levels of the GLP-1 degrading enzyme DPP-4 were lowered by -118.9 ± 56.15 ng/mL (*p*<0.05) (**Figure 3A**) following treatment with exenatide. There was no difference in DPP-4 following placebo treatment (**Figure 3B**). DPP-4 did not change following ingestion of glucose (data not shown).

**Figure 4:**
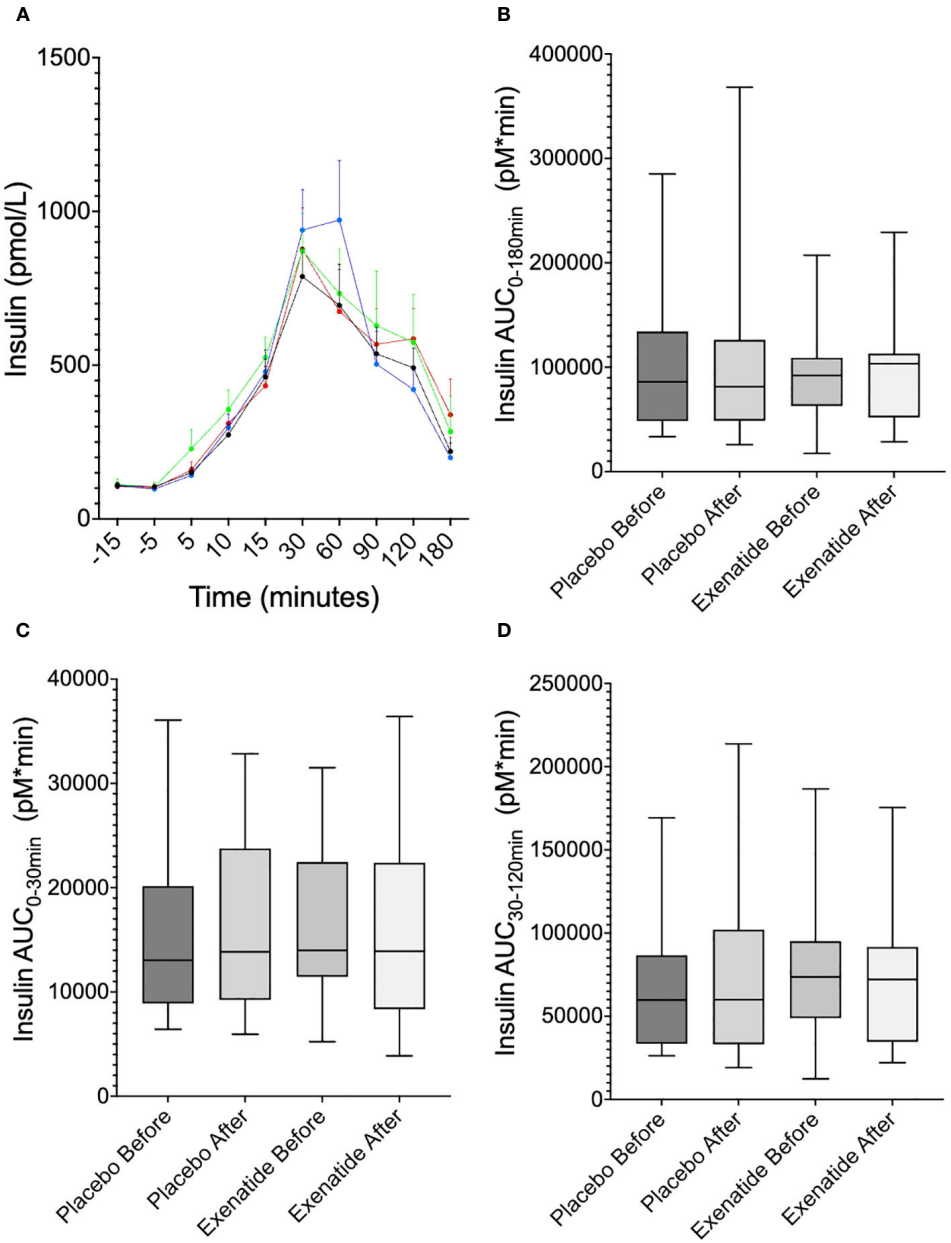
Plasma insulin levels. **(A)** shows insulin levels during the OGTT at randomization (blue) and at 6 months (black), before placebo (red) and after six months of placebo (green), **(B)** area under the curve (AUC)_0-180 min_, **(C)** AUC_0-30min_, **(D)** AUC_30-120 min_. Means ± SEM are shown.

**Figure 5:**
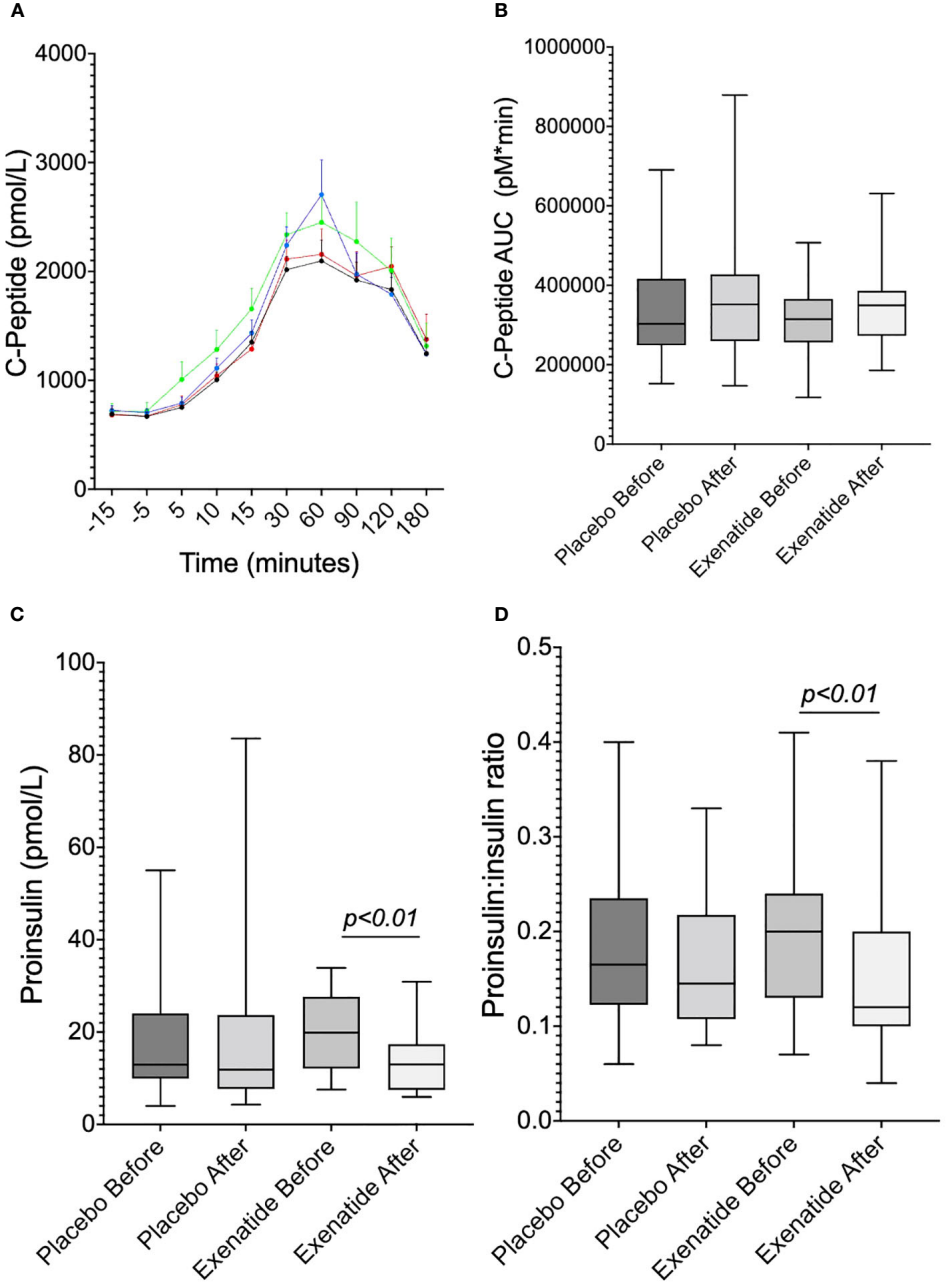
Illustrates **(A)** the C-peptide levels during the OGTT and **(B)** AUC_0-180min_. **(C)** illustrates proinsulin at fasting, and **(D)** the proinsulin-to-insulin ratio at fasting. Means ± SEM are shown.

Neither the curve (**Figure 4A**) nor the AUC (**Figure 4B**) of plasma insulin during OGTT changed following exenatide treatment. In both groups before and after treatment, insulin peaked at 30 minutes but was prolonged and still present at 60 minutes after exenatide treatment (**Figure 4A**). Neither first nor second phase insulin, seen as AUC_0-30min_ (**Figure 4C**) and AUC_30-120min_ (**Figure 4D**), were changed following treatment with exenatide. Similarly, exenatide treatment induced no difference in plasma C-peptide levels during OGTT (**Figures 5A**, **B**). Fasting proinsulin levels decreased in the exenatide group (mean difference -4.96 ± 1.41 pmol/L, *p*<0.01) but not in the placebo group (**Figure 5C**). The proinsulin-to-insulin ratio was lowered following treatment with exenatide, mean difference -0.038 ± 0.012 (*p*<0.01) (**Figure 5D**).

**Figure 6:**
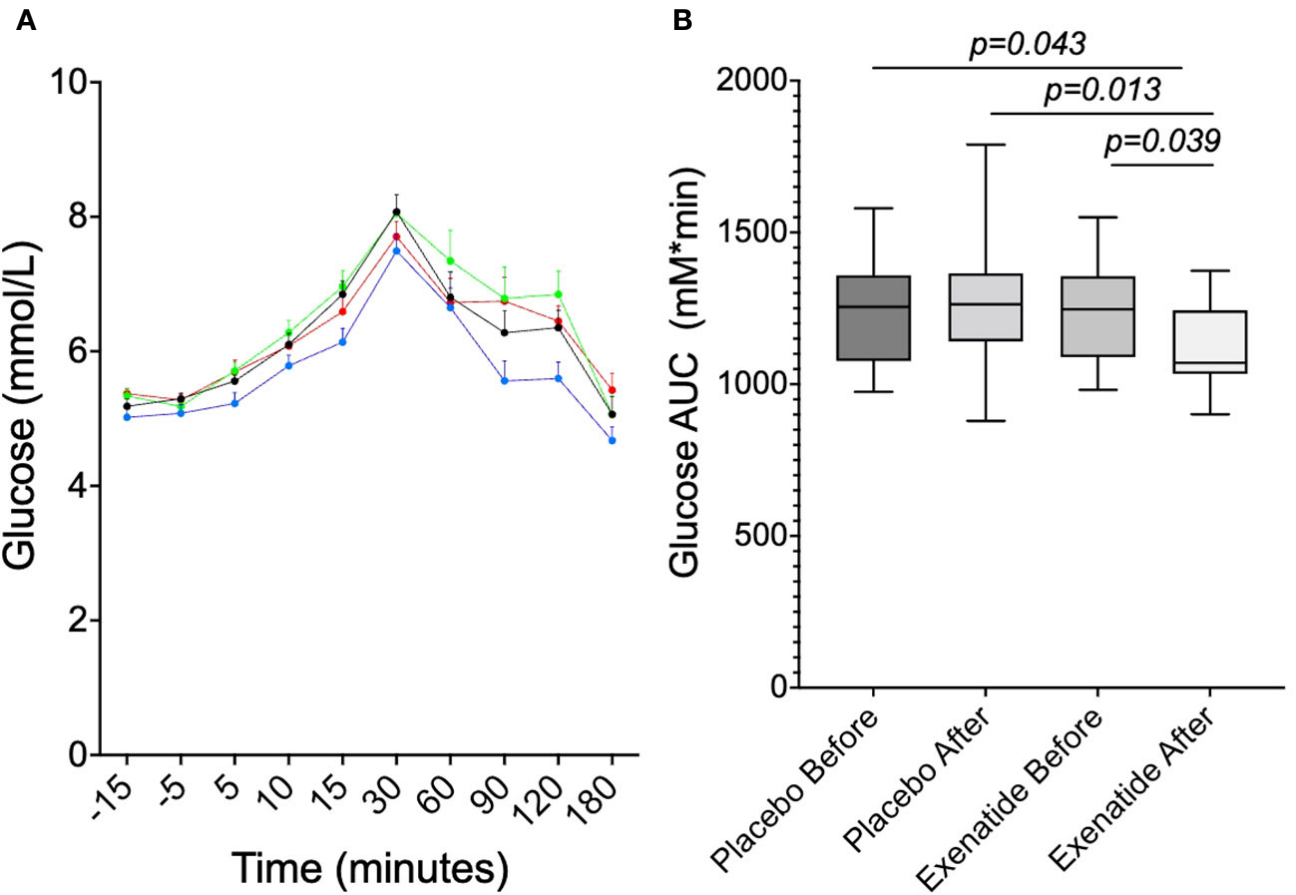
Plasma glucose concentrations during OGTT at randomization and at 6 months. **(A)** shows the glucose curve for before exenatide (black, *P*<0.001vs exenatide after), after six months of exenatide (blue), before placebo (red, *P*<0.0001vs exenatide after) and after six months of placebo (green, *P*<0.0001vs exenatide after). **(B)** shows the glucose area under the curve (AUC). Means ± SEM are shown.

Finally, the plasma glucose levels during the OGTT were analyzed. When comparing curves, the glucose values were significantly (p<0.001) lower after treatment with exenatide compared to before treatment, as well as compared to placebo before (*p*<0.0001) and after (*p*<0.0001) six months (**Figure 6A**). Furthermore, when comparing the AUC during the OGTT (AUC_0-180min_) of glucose, it was significantly smaller after treatment as compared to the three other groups (*p*<0.05 for all three) (**Figure 6B**).

## 4 Discussion

Exenatide has previously been shown to lower BMI (13, 22) and improve 2-hour glucose in adolescents with obesity (13). In the present study, we demonstrate that treatment with the GLP-1RA exenatide in adolescents with obesity improves glucose homeostasis, lowers circulating DPP-4 and proinsulin, and slightly increases glicentin, without affecting plasma levels of endogenous total GLP-1, glucagon, insulin or C-peptide.

Endogenous active GLP-1 has an *in vivo* half-life of a couple of minutes, due cleavage by DPP-4 yielding the inactive metabolite GLP-1 (9–36) amide (23). This metabolite is mainly cleared renally (23, 24), but peripheral tissue extraction could also play a part in the clearance (23). The lowering of DPP-4 levels observed in the present study is in accordance with the maintained GLP-1 levels and may be specific to GLP-1RA exenatide, since liraglutide, with its beneficial metabolic effects, has been shown to suppress endogenous total GLP-1 in adults with obesity (18). In another study, where adult patients with early T2DM were treated with liraglutide, enhanced endogenous GLP-1 response was observed (17). Notably, different ELISAs were used in these studies. Different GLP-1 ELISAs vary considerably in their specificity and sensitivity, which should be taken into account when comparing data from different studies (25, 26).

The present finding that exenatide lowers DPP-4 levels is important because circulating DPP-4 levels are elevated in obesity, regardless of glucose tolerance (21, 27). A serine protease, DPP-4 is responsible for rapidly inactivating GLP-1 (28) and various other substrates (21). The enzyme originates, in part, from adipocytes, especially those in visceral adipose tissue, as well as from macrophages invading adipose tissue (27). Clinically, inhibition of DPP-4 using gliptins is a common strategy for the treatment of T2DM, with beneficial effects on glycemic control (29). The observed decrease in DPP-4 could be a result of weight loss following exenatide treatment. Indeed, one study on children with obesity reported an association between weight loss and decreased DPP-4 in plasma (30). On the other hand, the reduction of circulating DPP-4 we see following exenatide treatment may be a novel off-target effect, adding to exenatide’s GLP-1 receptor binding effect. Even though DPP-4 is a ubiquitously expressed transmembrane enzyme (31), it is also known as CD26, as it partly originates from immune cells (32). GLP-1RA have known anti-inflammatory effects (33) and hence the reduction of DPP-4 could perhaps be a part of an overall reduction of inflammation following treatment with GLP-1RA.

Although glicentin showed considerable variability, we observed, overall, a slight increase of glicentin levels. This indicates a shift towards using the *GCG*-gene in the direction of GLP-1 over glucagon after exenatide treatment, although no significant reduction in glucagon was observed in this study. It has previously been reported that as glucose tolerance deteriorates in adolescents with obesity, glicentin levels are lowered (5), Exenatide may prevent this, and aid in maintaining healthy levels of glicentin. In a study on adults with obesity, liraglutide suppressed glicentin (18).

In the present study we also observed that the GLP-1RA exenatide lowered circulating levels of proinsulin in adolescents with obesity. Proinsulin is the precursor of insulin (34). It accounts for approximately 40% of the total protein production of the pancreatic β-cell, and increases as a result of a higher insulin demand from the periphery (35). Only a small percentage of proinsulin is secreted to the circulation (35), where it can be measured as a marker of β-cell stress (36). Following exenatide treatment, fasting proinsulin decreased, indicating a lower demand of insulin from the periphery and less stress on the β-cells. Indeed, a reduced proinsulin-to-insulin ratio has been reported following treatment with liraglutide in adults (37). Here, we report a similarly reduced proinsulin-to-insulin ratio following treatment with exenatide in adolescents. Neither insulin nor C-peptide were affected by exenatide treatment in this study. Fasting, first phase, second phase, and total insulin AUC were all unchanged following treatment. This is in agreement with previous findings of exenatide administration twice daily in adolescents with obesity having no effect on fasting insulin (22). While insulin secretion has previously been shown to increase during an intravenous glucose tolerance test after treatment with liraglutide in adults with T2DM (38), it is worth noting that different GLP-1RA have different documented effects on insulin secretion in adults. Short-acting GLP-1RA are shown to lower the insulin secretion, whereas long-acting GLP-1RA have been shown to enhance the insulin secretion (39). Insulin is an anabolic hormone, of great importance during puberty. Puberty induces a physiological insulin resistance (IR) (40). Thus, the effects of exenatide on insulin levels in adolescents should be interpreted with caution. The fact that exenatide treatment had no effect on insulin levels, but improved glucose tolerance and, as previously shown had an effect on weight and BMI-SDS (13), makes it an interesting candidate for the treatment of obesity during adolescence without the risk of altering insulin during growth.

Impaired glucose tolerance (IGT) and T2DM are evident risks for the individual with obesity (41), especially if the overweight or obesity is maintained throughout puberty (42). Exenatide clearly improved the glycemic control in this trial, which may reduce the risk of developing T2DM throughout adolescence and further during adulthood.

Weight loss maintenance is difficult, and particularly difficult in adolescents (43). It has been suggested that this may be due to developmental aspects, such as a less developed executive function (44). Indeed, weight loss maintenance therapy for obesity using GLP-1RA showed a significant effect on weight loss maintenance in adults using liraglutide (45), but not in adolescents using exenatide (43), underlining the difficulties of treating pediatric obesity. Unsatisfying adherence to medical treatment is a known problem in clinical obesity (46), hence weekly injections is theoretically a preferable treatment regimen over daily administration.

When comparing exenatide to other GLP-1RA, such as liraglutide or semaglutide, it is important to note that these drugs have different molecular structures, dosing regimens, and maximum plasma concentrations (47). Indeed, the analogs have substantially different effects on glycemic and body-weight outcomes (47). Whether our results on proglucagon-derived peptides differ from those with liraglutide (18) due to differences in the molecular structures of liraglutide and exenatide (47), the different study designs (OGTT vs mixed meal), or the fact that this study was conducted in adolescents as opposed to adults, is not possible to determine based on the present study’s design and should be further investigated. In addition, the effect of the new GLP-1 and GIP dual agonist tirzepatide (48) and the combination drug of orlistat and acarbose (49) would be immensely interesting to investigate in adolescents with obesity.

Since the per-protocol approach was used to analyze the data in this sub-study, we cannot be sure that the different groups are still comparable due to unidentified reasons (19), and hence the advantages of randomization might be reduced. However, at baseline the per-protocol adjusted groups did not differ significantly in age, sex, BMI, pubertal status or glucose tolerance. In addition, the drop-out rate did not differ between the groups, lessening the concerns (50). Further, the Combat-JUDO RCT (13) was not powered for this sub study, and the relatively small number of participants needs to be taken into consideration. Hence, larger studies are needed in the future to confirm the results.

In conclusion, weekly s.c. injections with 2 mg of exenatide maintains endogenous total GLP-1 levels and lowers circulating DPP-4 levels. This adds an argument in favor of using exenatide in the treatment of pediatric obesity.

## Data availability statement

The raw data supporting the conclusions of this article will be made available by the authors, without undue reservation.

## Ethics statement

The studies involving humans were approved by EudraCT No: 2015-001628-45; EC Sweden: Dnr 2015/279; EC Austria: 415-E/1544/20-2014. The studies were conducted in accordance with the local legislation and institutional requirements. Written informed consent for participation in this study was provided by the participants’ legal guardians/next of kin.

## Author contributions

RS: Conceptualization, Formal Analysis, Investigation, Methodology, Project administration, Visualization, Writing – original draft, Writing – review & editing. SC: Conceptualization, Formal Analysis, Investigation, Methodology, Visualization, Writing – original draft, Writing – review & editing. QW: Formal Analysis, Writing – review & editing. BA: Investigation, Methodology, Supervision, Writing – review & editing. HM: Conceptualization, Data curation, Formal Analysis, Funding acquisition, Investigation, Methodology, Project administration, Resources, Supervision, Validation, Visualization, Writing – original draft, Writing – review & editing. AC: Supervision, Writing – review & editing. HK: Formal Analysis, Funding acquisition, Investigation, Writing – review & editing. IC: Conceptualization, Formal Analysis, Investigation, Methodology, Project administration, Validation, Writing – review & editing. EG: Writing – review & editing. KM: Conceptualization, Funding acquisition, Investigation, Methodology, Project administration, Writing – review & editing. JG: Project administration, Writing – review & editing. VH: Project administration, Writing – review & editing. DW: Conceptualization, Data curation, Formal Analysis, Funding acquisition, Investigation, Methodology, Project administration, Resources, Supervision, Validation, Visualization, Writing – review & editing. AF: Conceptualization, Data curation, Formal Analysis, Funding acquisition, Investigation, Methodology, Project administration, Resources, Supervision, Validation, Visualization, Writing – review & editing. PB: Conceptualization, Data curation, Formal Analysis, Funding acquisition, Investigation, Methodology, Project administration, Resources, Software, Supervision, Validation, Visualization, Writing – original draft, Writing – review & editing.
